# Exome sequencing-based identification of *DNAAF1* variants implicated in male infertility and primary ciliary dyskinesia

**DOI:** 10.3389/fmolb.2026.1769803

**Published:** 2026-04-21

**Authors:** Baoqiong Liao, Wuming Xie, Mei Shuai, Lin Xiao, Jungao Huang, Ying He, Shuwen He

**Affiliations:** 1 Ganzhou Maternal and Child Health Hospital, Ganzhou, Jiangxi, China; 2 Ganzhou People’s Hospital, Ganzhou, Jiangxi, China; 3 Department of Chemistry and Molecular Biology, Faculty of Science, Gothenburg University, Gothenburg, Sweden; 4 Loke Centre for Trophoblast Research, Department of Physiology, Development, and Neuroscience, University of Cambridge, Cambridge, United Kingdom

**Keywords:** DNAAF1, exome sequencing, primary ciliary dyskinesia, sperm flagella, variant interpretation

## Abstract

Primary ciliary dyskinesia (PCD) is a rare, autosomal recessive disorder caused by impaired cilia and flagella function. Despite advances in molecular diagnostics, pathogenic variants remain to be detected in a subset of clinically diagnosed individuals. In the present case, abdominal ultrasonography revealed situs inversus of the liver and spleen, and chest X-ray demonstrated dextrocardia. Semen analysis showed markedly reduced sperm motility, consistent with ciliary dysfunction, and the patient exhibited additional clinical features characteristic of PCD. Exome sequencing (ES) revealed biallelic variants in dynein axonemal assembly factor 1 (DNAAF1) (NM_178452.6), including a missense variant, c.524T>C (p.Leu175Pro), and a nonsense variant, c.1462C>T (p.Arg488*). Segregation analysis was performed in the available family members and confirmed that each parent carried one of the variants in a heterozygous state. Bioinformatic predictions supported the pathogenic potential of identified variants, suggesting that they likely underlie the ciliary defects observed in the affected individual. Taken together, these findings implicate previously reported *DNAAF1* variant c.1462C>T and newly identified variant c.524T>C in PCD associated with male infertility. The predicted structural perturbation in *DNAAF1* protein structure is likely to impair dynein arm assembly, leading to loss of ciliary motility and the resultant clinical phenotype.

## Introduction

Cilia are hair-like protrusions of the plasma membrane and represent essential cellular organelles with diverse functions. They play critical roles in airway mucus clearance (Bustamante-Marin and Ostrowski, 2017), cerebrospinal fluid circulation (Faubel et al., 2016; Kumar et al., 2021), leftward extraembryonic fluid flow in the embryonic node (Hirokawa et al., 2006; Shinohara et al., 2012), and movement of the sperm (Yuan et al., 2019) and fertilized ovum (Suarez and Wolfner, 2021; Yuan et al., 2021). The majority of vertebrate cell types express a single immotile sensory primary cilium. However, multiple specialized motile cilia are present on differentiated epithelial cell-types of the brain, respiratory tract, sperm and fallopian tubes. Dysfunction of motile cilia has emerged as the cause of severe defects collectively termed primary ciliary dyskinesia (PCD; MIM 244400), a rare disorder affecting approximately 1 in 10,000–20,000 live births with an estimated incidence of 1 in 7,500 to 1 in 30,000 (Mianné et al., 2018; Lucas et al., 2020; Wee et al., 2024). PCD is characterized by chronic respiratory tract infection (Tilley et al., 2015), situs inversus (Pennekamp et al., 2015) and hydrocephalus (Munch et al., 2025), wherein infertility is a frequent comorbidity. To date, more than 50 genes have been implicated in PCD, with approximately 15–20 of these specifically associated with significant fertility defects, particularly affecting male reproductive function (Ji et al., 2017; Roostaei et al., 2025).

Infertility is a global health problem that affects 15% of couples, and 20%–30% of cases are related to men (Agarwal et al., 2015; Mehra et al., 2018). Male infertility represents a highly heterogeneous pathological condition that affects approximately 7% of the male population (Bracke et al., 2018). Male infertility is generally recognized only after the onset of puberty, systematic assessment of sperm parameters is rarely performed in individuals with known PCD (Jayasena and Sironen, 2021). Consequently, existing documentation of male reproductive outcomes remains incomplete, and the specific contributions of PCD-associated genes to human flagellar function are still insufficiently defined.

The axoneme, the core of motile cilia and flagella, consists of nine peripheral outer doublet microtubules surrounding a central microtubule pair. Along each doublet, there are inner and outer dynein arms that hydrolyze ATP to power ciliary movement, radial spokes that modulate ciliary beating (Castleman et al., 2009), and a spoke-associated dynein regulatory complex (Heuser et al., 2009). Axonemal dynein is attached to the doublet microtubules in two main continuous rows known as the outer dynein arm (ODA) and the inner dynein arm (IDA). ODAs and IDAs are connected to the α-microtubule of each peripheral doublet for driving the sliding of the microtubule doublets (Pazour et al., 2006). While the composition of dynein arms varies among species, human respiratory cilia possess ODAs composed of a globular head domain containing the heavy chains HCβ (*DNAH11*/*DNAH9*) and HCγ (*DNAH5*), an intermediate domain formed by *DNAI1* and *DNAI2*, and a docking complex comprising *CCDC114*, *CCDC151*, *ARMC4*, and *TTC25*, which secures the ODA to the adjacent microtubule doublet (Fliegauf et al., 2005; Nicastro et al., 2006). Variants in genes encoding dynein assembly factors, radial spoke components, components of the ODA and dynein regulatory complex have all been shown to cause PCD with laterality defects and male infertility (Loges et al., 2008; Ji et al., 2017; Whitfield et al., 2019; He et al., 2023).

*DNAAF1* (MIM: 613193, known as *LRRC50*) is located on chromosome 16q24.1 and contains 12 exons, encoding a protein containing 725 amino acids. DNAAF1, the human ortholog of Chlamydomonas ODA7, functions as a dynein assembly factor, and variants in *ODA7*/*DNAAF1* impair dynein arm assembly, leading to reduced ciliary beat frequency (Freshour et al., 2007). Mutants of *dnaaf1*,the zebrafish ortholog of human DNAAF1, exhibit classic motile cilia phenotypes, including pronephric cysts, and randomized heart jogging and visceral laterality defects in over 50% of embryos (Sullivan-Brown et al., 2008; van Rooijen et al., 2008). The *DNAAF1* mutants have ultrastructural abnormalities of the dynein arms (lacking either ODA, or both IDA and ODA) and outer microtubule misalignment (Basten et al., 2013). In human respiratory epithelia, *DNAAF1* variants result in the absence of key dynein subunits from the ciliary axoneme, confirming its essential role in dynein preassembly (Loges et al., 2009). Currently, the studies reported *DNAAF1* variants, primarily focusing on the classic PCD phenotypes including situs invervus (Loges et al., 2009; Zhou et al., 2020), bronchiectasis (Ito et al., 2024) and neural tube defects (Miao et al., 2016). In addition, one study identified a *DNAAF1* variant in a patient with seminoma (Basten et al., 2013). However, evidence linking *DNAAF1* variants to reproductive dysfunction remains limited and inconclusive.

In this study, we investigated a patient with primary ciliary dyskinesia and male infertility to identify the underlying genetic cause and characterize the functional consequences of *DNAAF1* variants. By combining exome sequencing, *in silico* structural modeling using AlphaFold, and functional assays in human cell lines, we aimed to elucidate how *DNAAF1* variants affect protein structure, stability, and dynein arm assembly. This work provides new insights into the genotype–phenotype correlations of *DNAAF1* and expands our understanding of its critical role in ciliary motility and male reproductive health.

## Materials and methods

### Ethical compliance

Informed consent was obtained from the patient prior to study enrollment. The research was approved by the Ethics Committee of Ganzhou Maternal and Child Health Hospital (Approval No. 2024-103, 15 November 2024). All procedures were performed in accordance with the ethical standards of the institutional and national research committees, as well as with the declaration of Helsinki.

### Sample collection and DNA extraction

Peripheral venous blood samples were taken from the proband and all available family members following the acquisition of informed consent from all participants. Genomic DNA was extracted using the QIAamp DNA Mini Kit for blood (Qiagen, Hilden, Germany), as previously described (Liao et al., 2025b; 2025a; Xie et al., 2025). DNA quality and integrity were assessed by agarose gel electrophoresis, and concentrations were measured using Qubit 2.0. A minimum of 1.5 μg of high-quality DNA from the proband was used for library preparation.

### Library preparation and exome sequencing

Exome sequencing (ES) was performed by Kangxu Diagnostics (Beijing, China). Genomic DNA was fragmented to 180–280 bp, followed by end repair, A-tailing, and adapter ligation with unique indexes. Libraries were pooled and hybridized to the NEXome Core Panel to capture the exons of over 20,000 genes. Captured fragments were PCR-amplified and quality-checked using Qubit quantification, Agilent 4150 insert size analysis, and qPCR. Libraries that passed the quality control (QC) were sequenced on the BGISEQ-T7 platform to an average depth of 100×, and coverage metrics, including the proportion of target bases ≥20×, were assessed. Raw sequencing data were generated in FASTQ format.

### Variant analysis

Sequencing data were converted from BCL to FASTQ using bcl2fastq (v2) and aligned to the human reference genome (hg19) with BWA (v0.7.15). SNVs and small insertions/deletions were identified using GATK (v3.6), while CNVs were analyzed with CODEX, XHMM (v1.0), and KSCNV (developed by Kangxu Diagnostics). Variants were annotated with ANNOVAR (v2016-02-01) for gene/transcript position, protein impact (PolyPhen2 (http://genetics.bwh.harvard.edu/pph2/), SIFT (https://sift.bii.a-star.edu.sg/index.html),MutationTaster(https://www.mutationtaster.org/)), disease associations (OMIM, HGMD, ClinVar), and population allele frequencies (1000G, ESP6500, gnomAD). Variant screening integrated clinical phenotypes, population and disease databases, and functional prediction tools, with extremely rare variants identified (c.524T>C: gnomAD_exome AF = 0.00000136809; c.1462C>T: AF = 0.0000219618). Variants were filtered and prioritized based on genomic location, type, rarity (MAF <1%), predicted functional impact, and known disease associations, then classified according to ACMG/AMP guidelines.

### Sanger sequencing verification

Candidate gene variants identified by exome sequencing in the proband were validated by Sanger sequencing. Gene sequences for the candidate variants were obtained from the GenBank database, and primers were designed using Primer Z (http://genepipe.ncgm.sinica.edu.tw/primerz/primerz4.do) and subsequently synthesized. PCR amplification of the candidate variant sites was performed using the following primers: *DNAAF1* F1: GGCAAAAACAAGGGTGACCG, *DNAAF1* R1: TCAGGGGAAGGTGATGGACA, *DNAAF1* F2: GGGGACAGAGAAACAAGGCA, and *DNAAF1* R2: GTCCCACAGAGACGTGAGTC. PCR products were verified on 1% agarose gel, purified, and subjected to Sanger sequencing on an ABI 3730 DNA analyzer. Sequencing results were analyzed and aligned to the reference sequences to validate variants and exclude potential false positives identified in prior next-generation sequencing.

### *DNAAF1* plasmid constructs

Full-length *DNAAF1* cDNA sequences, including the wild-type (WT) and the variants c.524T>C and c.1462C>T, were synthesized and cloned into the pCDNA3.1 expression vector containing an N-terminal His tag, generating pCDNA3.1-*DNAAF1*-WT, pCDNA3.1-*DNAAF1*-mut1, and pCDNA3.1-*DNAAF1*-mut2, respectively. The primers used for the synthesis of these cDNAs are listed in Supplementary Table S1. The WT and mutant cDNAs were inserted into the pCDNA3.1 vector using the restriction enzymes KpnI and XbaI, followed by ligation with T4 DNA ligase (Thermo Fisher Scientific, USA). Recombinant plasmids were isolated from individual bacterial colonies and screened by restriction enzyme digestion to verify the presence of inserts of the expected size. The sequence integrity and correctness of all constructs were further confirmed by sequencing.

### Cell culture and transfection

HEK293 cell lines (American Type Culture Collection, ATCC) were cultured in Dulbecco’s Modified Eagle Medium (DMEM) sourced from GIBCO Life Technologies. The medium was supplemented with 10% fetal bovine serum (FBS) from Invitrogen, 100 U/mL penicillin-streptomycin (GIBCO Life Technologies), and 2.5 μg/mL Plasmocin (InvivoGen). Cells were incubated at 37 °C in a humidified environment with 5% CO2. Transfections were performed using Lipofectamine 2000 (Invitrogen, Burlington, ON, Canada), followed by a 48-h incubation prior to western blot analysis.

### Western blot

Cell samples were collected and lysed in sodium dodecyl sulfate (SDS) loading buffer, followed by heating at 100 °C for 5 min. Proteins were separated by SDS-PAGE and subsequently transferred onto polyvinylidene fluoride (PVDF) membranes. The membranes were blocked with Tris-buffered saline (TBS) containing 0.1% Tween-20% and 5% skimmed milk for 1 h at room temperature to minimize nonspecific binding. Membranes were then incubated overnight at 4 °C with primary antibodies: His-Tag monoclonal antibody (Proteintech, Cat. No. 66005-1-Ig, 1:1000) and GAPDH rabbit monoclonal antibody (Abclonal, Cat. No. A19056, 1:1500). Following three washes with TBST (10 min each), membranes were incubated for 1 h at room temperature with HRP-conjugated secondary antibodies: goat anti-mouse IgG (H + L) (Thermo Fisher, Cat. No. 31430) and goat anti-rabbit IgG (H + L) (Thermo Fisher, Cat. No. 31460), both diluted 1:10,000. After three additional washes with TBST, protein signals were visualized using an enhanced chemiluminescence (ECL) detection substrate.

### Protein structure prediction

The protein sequence with 725 amino acid residues of DNAAF1 was downloaded from uniprot web (UniProt accession Q8NEP3, https://www.uniprot.org/). Wild-type and mutant DNAAF1 proteins, corresponding to c.524T>C (p.Leu175Pro) and c.1462C>T (p.Arg488*), were modeled in 3D using the AlphaFold web server https://golgi.sandbox.google.com/about) (Abramson et al., 2024a). The best model was selected based on pLDDTs prediction scores. The higher the score, the more confident is the structure. Prediction models editing was performed and visualized using PyMOL program (https://pymol.org/).

### Bioinformatics analysis

The protein-protein interaction (PPI) network associated with DNAAF1 was constructed using STRING (https://string-db.org/). The amino acid sequence of the DNAAF1 protein, comprising 725 residues, was retrieved from the UniProt database (https://www.uniprot.org/). This sequence was used as the input query for STRING, where the parameters were set to ensure high-confidence interactions were captured. The confidence score threshold was adjusted to include only interactions supported by strong evidence such as experimental data, co-expression, and co-occurrence analyses.

## Results

### Heterozygous variants of *DNAAF1* identified in a patient with male infertility and PCD

**FIGURE 1:**
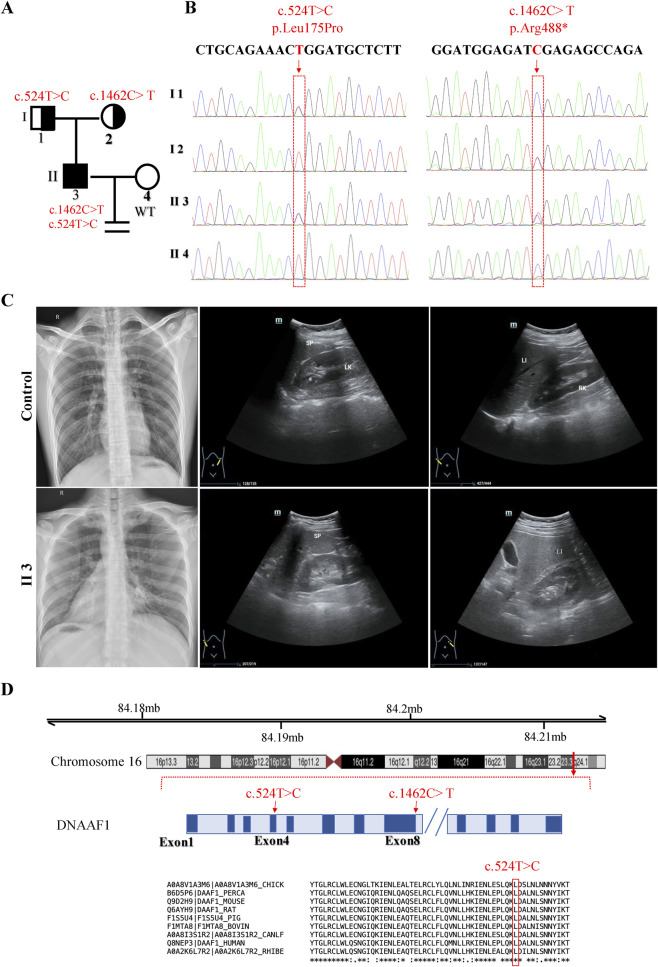
Identification of *DNAAF1* Variants in a Patient with Primary Ciliary Dyskinesia and Male Infertility. **(A)** Pedigree and Segregation analysis of *DNAAF1* variants in family. **(B)** The identified variants were confirmed by Sanger sequencing. **(C)** Abdominal ultrasonography and chest computed tomography images of the affected individual (II2) and a control. R, right; L, left; Sp, spleen; LK, left kidney; LI, liver; RK, right kidney. **(D)** Approximate locations of the identified variants and evolutionary conservation of the corresponding amino acids. Multiple sequence alignment of human *DNAAF1* with orthologues from other species is shown.

The affected individual (II3-4) presented to the hospital for assisted reproductive technology (ART) treatment due to severely reduced sperm concentration, motility, and overall semen quality, consistent with profound male factor infertility. Exome sequencing of the proband identified two heterozygous variants in DNAAF1: c.524T>C, a previously unreported missense variant, and c.1462C>T, a variant previously reported in seminoma (Basten et al., 2013). Sanger sequencing confirmed these variants and demonstrated that each parent carried one heterozygous allele, consistent with compound heterozygosity in the proband (Figures 1A,B). Abdominal ultrasonography and chest computed tomography (CT) revealed situs inversus, including complete reversal of the heart, spleen, and liver, whereas a healthy control exhibited normal organ positioning (Figure 1C). The c.524T>C variant is a missense variant located in exon 4, while the c.1462C>T variant is a nonsense variant located after exon 8, predicted to produce a truncated protein. Multiple sequence alignment showed that the affected residue corresponding to c.524T>C is highly conserved across species, highlighting its likely functional importance (Figure 1D). Collectively, these clinical observations, together with the identified *DNAAF1* variants, provide a genetic and phenotypic explanation for the patient primary ciliary dyskinesia with male infertility and laterality defects.

### Predicted structural and functional consequences of *DNAAF1* variants

**FIGURE 2:**
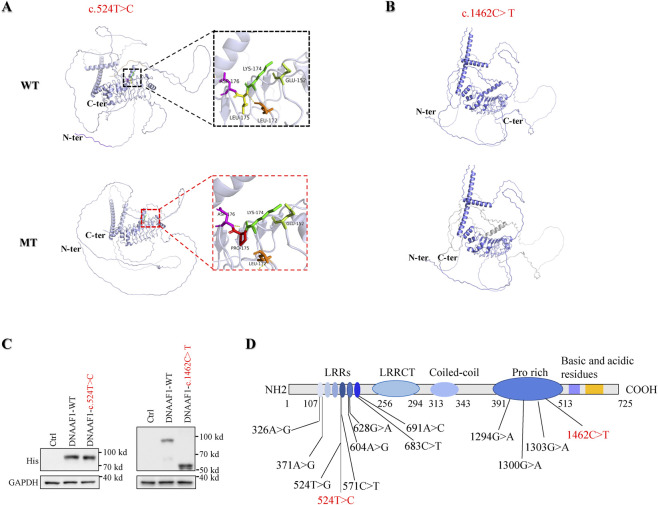
Predicted structural consequences of *DNAAF1* variants. **(A)** Predicted three-dimensional structure of the wild-type DNAAF1 protein. Leu-172 and Leu-175 form hydrogen bond interactions. The c.524T>C variant changes Leu-175 to Pro-175, disrupting the original hydrogen bond without forming new ones, leading to a local alteration in charge distribution. **(B)** Predicted structure of the c.1462C>T mutant DNAAF1 protein, resulting in a truncated protein comprising 488 of the 725 amino acids of the mature protein. **(C)** Expression of DNAAF1 WT and mutant proteins in HEK293 cells. *GAPDH* is used as a loading control. Protein expression of DNAAF1 c.524T>C was comparable to the wild type, whereas DNAAF1 c.1462C>T exhibited a truncated protein with a lower molecular weight. **(D)** Schematic representation of *DNAAF1* showing the localization of the identified variants within functional domains. DNAAF1 contains leucine-rich repeats (LRRs), a leucine-rich repeat C-terminal domain (LRRCT), a proline-rich region, and coiled-coil domains. The c.524T>C variant is located within the LRRs, while the c.1462C>T variant is situated in the proline-rich region, indicating their potential impact on protein structure and function.

To investigate the potential structural impact of the identified *DNAAF1* variants, three-dimensional protein structures were predicted using the AlphaFold web server (https://golgi.sandbox.google.com/about) (Abramson et al., 2024b), which generates accurate structural models based on amino acid sequence information. The predicted structure of the wild-type *DNAAF1* protein revealed that Leu-172 and Leu-175 form stabilizing hydrogen bonds, maintaining the integrity of the leucine-rich repeat (LRR) domain. The c.524T>C missense variant substitutes Leu-175 with proline, disrupting the hydrogen bond network and locally altering the charge distribution, which may affect domain stability and protein–protein interactions (Figure 2A). In contrast, the c.1462C>T variant introduces a premature stop codon, resulting in a truncated protein of 488 amino acids, with loss of critical C-terminal domains required for dynein arm and radial spoke interactions (Figure 2B). These AlphaFold-based structural predictions suggest that c.524T>C may cause subtle conformational perturbations, whereas c.1462C>T likely induces severe structural disruption, consistent with a loss-of-function effect.

### *DNAAF1* mutant protein expression analysis

The c.1462C>T variant in DNAAF1 is a nonsense variant predicted to generate a truncated protein due to premature termination of translation. To determine whether these mutant alleles could produce stable, intact DNAAF1 protein, we constructed expression plasmids carrying either wild-type or mutant *DNAAF1* sequences and transiently transfected them into HEK293T cells. Protein expression levels were subsequently evaluated by western blot using an anti-His antibody specific to the recombinant proteins. The results demonstrated that the c.1462C>T mutant exhibited markedly reduced molecular weight relative to WT, consistent with the predicted truncation and potential instability of the protein. In contrast, the c.524T>C missense variant did not substantially affect protein expression (Figure 2C) (Supplementary Figure S1). Domain mapping showed that c.524T>C resides within the leucine-rich repeats (LRRs), whereas c.1462C>T lies in the proline-rich region, indicating potential disruption of domain-specific functions contributing to defective dynein arm assembly and the observed ciliary dysfunction. (Figure 2D).

### Genotype-phenotype relationship in *DNAAF1*

To investigate the factors underlying phenotypic variability in *DNAAF1*-associated PCD and infertility, we systematically reviewed all reported *DNAAF1* variants and analyzed their associated clinical features. Reported pathogenic and likely pathogenic variants (Table 1) distributed across key functional domains of the *DNAAF1* protein, including the leucine-rich repeat (LRR) domains and the proline-rich region. To better understand genotype-phenotype correlations, the reported clinical data on *DNAAF1* variants were collected, including variants c.811T and c.1349_1350insC (Loges et al., 2009), c.691A > C (Miao et al., 2016), c.571C > T (Hartill et al., 2018), c.524T>G (Duquesnoy et al., 2009), c.943A>T, c.3G>A, c.124 + 1G>C, c.509delG and c.943A>T (Zhou et al., 2020), as well as c.86delG (Ito et al., 2024) (Table 2). *DNAAF1* variants are associated with a spectrum of PCD phenotypes, most commonly involving respiratory dysfunction.*DNAAF1* variants are also linked to the laterality defects, including totalis and partial situs inversus, as well as certain congenital heart defects, which could be detected during pregnancy. These findings further support *DNAAF1* as a causative gene in PCD with infertility and emphasizes the various clinical symptoms caused by different variant types.

**TABLE 1:** Mutations described in the *DNAAF1* gene (from the Human Gene Mutation Database).

Mutation	HGMD access ID	Amino acid change	Location on DNAFF1	Disease	PMID	Pathogenicity
326A>G	CM2227736	Asn-Ser	LRR1	Rett syndrome	36157478	Pathogenic
371A>G	CM1611688	Asn-Ser	LRR1	Neural tube defects	27543293	Pathogenic
524T>G	CM099261	Leu-Arg	LRR4	Primary ciliary dyskinesia	19944405	Likely pathogenic
524T>C	ND	Leu-Pro	LRR4	Primary ciliary dyskinesia	ND	Likely pathogenic
571C>T	CM182762	Leu-Phe	LRR4	Heterotaxy	29228333	Pathogenic
604A>G	CM1618864	Met-Val	LRR5	Multiple congenital anomalies	26633542	Pathogenic
628G>A	CM2077928	Val-Met	LRR5	Developmental disorder	33057194	Pathogenic
683C>T	CM2125649	Ser-Leu	LRR6	Primary ciliary dyskinesia	34556108	Pathogenic
691A>C	CM1611689	Lys-Gln	LRR6	Neural tube defects	27543293	Pathogenic
1294G>A	CM129169	Glu-Lys	Pro rich	Primary ciliary dyskinesia	22499950	Pathogenic
1300G>A	CM2219220	Gly-Arg	Pro rich	Primary ciliary dyskinesia	35804324	Pathogenic
1303G>A	CM1910538	Asp-Asn	Pro rich	Primary ciliary dyskinesia	31213628	Pathogenic
1462C>T	ND	Arg-*	Pro rich	Primary ciliary dyskinesia	ND	Likely pathogenic

ND, Not Determined.

**TABLE 2:** Clinical data of all DNAAF1 mutations in patients with primary ciliary dyskinesia.

Description	This study	Duquesnoy et al. (2009)	Tomas et al. (2009)	Miao et al. (2016)	Hartill et al. (2018)	Zhou et al. (2020)	Ito et al. (2024)
Mutation	c.524T>C, c.1462C>T	c.524T>G	c.811T, c.1349_1350insC	c.691A > C	c.571C > T	c.3G>A(1), c.124+1G>C(1), c.509delG(1), c.943A>T(2)	c.86del
Amino acid change	p.Leu175Pro, p.Arg488*	p.Leu175Arg	p.Arg271* p.451AlafsX5	p.Lys231Gln	p.Leu191Phe	p.Met-Ile, IVS1 ds G-C +1 p.Glu126Lysfs × 35 p.Lys315×	p.Gly29ValfsTer60
GnomAD frequencies	0.00000136809, 0.0000219618	Unknown	6.20e-7, unknown	6.19522645411258E-0	6.20036606961275E-07	1.89775647229845E-06, 6.4635893087062E-07, 1.85945726161448E-06	6.43086816720257E-07
Number of case	1	2	1	1	1	2	1
Age at dignosis (years old)	34	Unknown	16	Gestational age 38 Week	Unknown	32(1), 37(2)	58
Gender	Male	Male	Unknown	Male	Male	Male(1), Female(2)	Female
Respiratory symptoms	-	bronchitis, sinusitis, bronchiectasis,	Bronchiectasis	Pulmonary lobe malformation	-	bronchiectasis, lung infections, sinusitis	Bronchiectasis
Heart disease	-	-	-	-	subpulmonary stenosis inlet ventricular septal defect	-	-
Situs Abnormalities	Spleen and liver inversus, dextrocardia	No	Situs inversus totalis	Unknown	Stomach, Spleen and liver inversus,dextrocardia	Situs inversus	-
Reproductive disorders	infertility	Not tested	Unknown	Unknown	Unknown	Infertility	Infertility
Hearing symptoms	-	Otitis	-	Unknown	-	-	-
Other Features	-	-	-	Equinovarus, Absence of skull, Horseshoe kidney	-	-	-
Clinical Imaging							
Ultrasound	+	Unknown	Unknown	Unknown	Unknown	Unknown	Unknown
Computed tomography scan	+	Unknown	+	Unknown	+	+	+
Electron microscopy	Not applicable	+	+	Unknown	+	+	+

(+), present; (–), absent.

* termination codon.

### Impact of *DNAAF1* variants on dynein arm assembly

**FIGURE 3:**
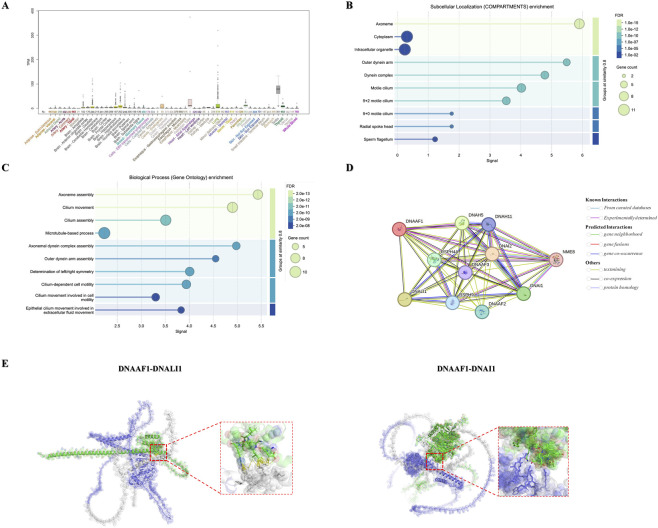
The predict effect of *DNAAF1* mutants on the dynein assembly. **(A)** GTEx data demonstrate that *DNAAF1* is expressed at an appreciable level in human tissue represented. **(B,C)** Subcellular localization and biological pathway of *DNAAF1*. **(D)** DNAAF1 protein-protein interactions using STRING software. **(E)** The impact of *DNAAF1* mutants on these protein-protein interactions, showing potential disruption of binding with ODA and IDA subunits. The purple structure represents the wild-type DNAAF1 protein, while the grey regions correspond to the portions absent in the c.1462C>T variant. The green structures depict *DNALI1*, *DNAI1*, and *DNAI2*.

*DNAAF1* is broadly expressed across multiple tissues, with relatively high expression in ciliated tissues like brain, cervix, fallopian tube, lung and testist (Figure 3A). The expression of *DNAAF1* was obtained from the GTEX (https://gtexportal.org/home/). Subcellular localization analysis indicates that *DNAAF1* is highly enriched in the axoneme, including both 9 + 0 non-motile cilia and 9 + 2 motile cilia, particularly within the outer dynein arms (ODA) and inner dynein arms (IDA) (Figure 3B). Gene ontology analysis of biological processes suggest that *DNAAF1* is involved in axoneme assembly, axonemal dynein arm assembly, and ODA assembly, all of which are critical for ciliary motility (Figure 3C). The data were derived from STRING database analysis (https://string-db.org/). To further investigate *DNAAF1* function, STRING database was employed to examine potential functional interactions, revealing that *DNAAF1* interacts with ODA subunits *DNAH5*, *DNAH11*, *DNAI1*, and *DNAI2*, as well as the IDA subunit *DNALI1* (Figure 3D).

Using AlphaFold3, we modeled the *DNAAF1*–*DNALI1* and *DNAAF1*–*DNAI1* complexes and found that the *DNAAF1*–*DNAI1* interface exhibits a substantially broader and more continuous surface-binding region compared with the *DNAAF1*–*DNALI1* interface (Figure 3E). The grey regions correspond to the portions that are absent in the c.1462C>T variant. The c.1462C>T (p.Arg488*) substitution was also predicted to markedly disrupt the local binding interface between *DNAAF1* and *DNALI1*. As indicated by the structural model with enlarged diagrams, this variant perturbed the surrounding residue geometry and eliminated stabilizing hydrogen-bond interactions normally formed within this region. As a consequence, the variant impaired the formation and stabilization of hydrogen bonds with neighboring residues that were essential for maintaining the *DNAAF1*–*DNALI1* and *DNAAF1*–*DNAI1* binding interface.

Together, these structural alterations strongly suggested that the c.1462C>T variant compromised *DNAAF1*’s ability to interact with its dynein arm subunits ultimately impairing the pre-assembly of both ODA and IDA components. This provided a mechanistic explanation for the defective dynein arm formation and ciliary dysfunction observed in patient with this variant.

## Discussion

Cilia are highly specialized cellular organelles whose architecture and motor machinery determine their biological roles. Among them, motile cilia exhibit the canonical “9 + 2” axonemal structure, composed of nine outer microtubule doublets surrounding a central pair (Mitchell, 2004; Loreng and Smith, 2017). Attached to the outer doublets are the outer dynein arms (ODAs) and inner dynein arms (IDAs), which function as ATP-dependent motor complexes that drive coordinated, rhythmic beating (Lin and Nicastro, 2018; Ishibashi et al., 2020; Leung et al., 2025). ODAs primarily regulate the frequency of ciliary motion, while IDAs are important for the waveform of the cilium (Brokaw and Kamiya, 1987; Kubo et al., 2021). Defects affecting one or both dynein arm components are key contributors to PCD (O’Callaghan et al., 2011; Reiter and Leroux, 2017), a genetically heterogeneous disorder with a well-characterized phenotype but no effective treatment (Mianné et al., 2018; Lee and Ostrowski, 2021).

In the present study, a patient with PCD and male infertility was found to carry two DNAAF1 variants: a nonsense variant previously reported in seminomas and a novel variant, expanding the spectrum of pathogenic variants.

In adult humans, *DNAAF1* expression is predominantly localized to tissues rich in motile cilia or flagella, including the respiratory epithelium, fallopian tubes, sperm and and specific regions of the brain that contain motile ependymal cilia Structural modeling is a valuable tool for predicting the impact of genetic variants on protein function. In other systems, *in silico* analyses have successfully revealed subtype-specific effects of protein-RNA interactions, such as for *HIV*-1 Tat–TAR RNA (Ronsard et al., 2017a; 2017b). By analogy, we assessed the predicted structural consequences of the *DNAAF1* c.524T>C and c.1462C>T variants. *DNAAF1* contains six conserved N-terminal leucine-rich repeats (LRRs), a coiled-coil region, a leucine-rich repeat C-terminal domain (LRRCT), and a non-conserved proline-rich domain (van Rooijen et al., 2008). *DNAAF1* is essential for the preassembly of IDAs and ODAs (Miao et al., 2016). Although its precise mechanism remains unclear, extensive evidence supports its crucial role in dynein arm assembly. Loss of *DNAAF1* function impairs the incorporation of ODA heavy chains (*DNAH5*, *DNAH9*), ODA intermediate chains (*DNAI2*), and IDA light intermediate chains into cilia, leading to their abnormal accumulation within the cytoplasm (Loges et al., 2009). Insilco structural predication in our studies revealed that *DNAAF1* directly interacted with *DNAI2* and *DNALI1*. The variants identified in affected individuals are likely disrupt these critical interactions, impairing ODA and IDA assembly and contributing to the severe ciliary motility defects, laterality abnormalities, and markedly reduced sperm motility observed in the patient. Functional consequences of protein variants are commonly assessed *in vitro*, as illustrated by HIV-1 Tat variants, which show subtype-specific effects on RNAi silencing and cell death (Ronsard et al., 2019b). Notably, Miao et al. (Miao et al., 2016) demonstrated that *DNALI1* labeling is absent in MDCK cells transfected with the *DNAAF1* p.Lys231Gln mutant, highlighting the critical role of *DNAAF1* in IDA assembly. The defective preassembly underlies the motility defects observed in patients with *DNAAF1* variants and highlights its essential role in dynein arm assembly and the maintenance of ciliary structure.

The patient exhibited hallmark laterality defects, including situs inversus of visceral organs and dextrocardia, reflecting defective nodal ciliary flow during embryonic development. Previous studies demonstrated that altered *DNAAF1* expression correlated with changes in the expression of left–right patterning genes and sonic hedgehog signaling-related genes, including Lefty1 (Meno et al., 1998) and Lefty2 (Saijoh et al., 2000) in NE-4C neuroectodermal stem cells (Miao et al., 2016). These collectively support that *DNAAF1* dysfunction perturbs left–right axis specification, thereby contributing to the development of laterality defects. In addition, severely reduced sperm motility in the patient was consistent with impaired flagellar axonemal function, further supporting an underlying ciliopathy.

The *DNAAF1* c.1462C>T variant produced a *DNAAF1* protein of decreased molecular weight in transfected HEK293T cells, consistent with its predicted truncating effect. In contrast, the c.524T>C missense variant exhibited protein expression levels comparable to the wild-type construct. Similarly, the c.524T>G variant, which affects the same nucleotide position but results in a different amino acid substitution. The clinical features observed in patients carrying the c.524T>G variant further support its pathogenic potential and reinforce the genotype–phenotype correlation in DNAAF1-associated PCD (Duquesnoy et al., 2009) (Table 2). Another missense variant, c.571C>T, has also been associated with a classic PCD phenotype characterized by male infertility (Hartill et al., 2018). Structural modeling indicated that this missense variant is located within the LRR4 domain, a region essential for *DNAAF1* function, as deletion of the LRR domain failed to rescue the zebrafish mutant phenotype (van Rooijen et al., 2008). This alteration may disrupt the cytoplasmic preassembly of dynein-arm complexes, consistent with interactions observed for other dynein subunits. Together, these findings indicated that the novel *DNAAF1* variants identified in this study probably resulted in loss of function and impaired motile ciliary activity. Studies of disease-associated genes in diverse populations, such as the characterization of CCR5 polymorphisms (Ronsard et al., 2019a), illustrate how population-specific genetic analyses can reveal novel variants that may influence disease susceptibility. Similarly, our study expands the known spectrum of *DNAAF1* variants in PCD patients.

Among the clinical manifestations of PCD, infertility is observed, with males being more severely affected due to impaired sperm motility. Notably, assisted reproductive technologies (ART) have enabled some patients, including the individual described in our study, to achieve successful pregnancies and healthy offspring. Patients have achieved successful outcomes following intracytoplasmic sperm injection (ICSI), suggesting that ICSI may be an effective option for enabling these patients to conceive. However, ICSI cycles are performed prior to genetic analysis in our study, underscoring the importance of variant screening in patients during ART treatment. Impaired motility of sperm flagella resulting from axonemal defects associated with *DNAAF1* variants can partially or completely abolish their ability to swim, ultimately leading to male infertility (Yw et al., 2014). In females, coordinated ciliary beating, together with muscular contractions, facilitates the transport of the oocyte through the fallopian tube to the uterus, and dysfunction in this process can contribute to subfertility (Lyons et al., 2006; Yuan et al., 2021). Nevertheless, compared with male patients, female individuals with PCD generally retain a higher likelihood of achieving spontaneous conception and fertility (Raidt et al., 2015; Newman et al., 2023). Literature review revealed two male and three female patients with *DNAAF1* variants exhibiting infertility (Table 2). Due to the limited number of reported cases, a definitive association between *DNAAF1* variants and female infertility remains to be well established.

Beyond ART, preclinical studies have explored potential therapeutic strategies targeting genetic related infertility. Injection of *dnaaf* mRNA into *dnaaf1*-deficient zebrafish has been shown to restore ciliary function (van Rooijen et al., 2008). Testicular mRNA delivery using lipid nanoparticles in a nonobstructive azoospermia mouse with testis-specific gene *Pdha2* variant restores spermatogenesis and enabled the production of viable offspring (Mashiko et al., 2025). Nevertheless, whether exogenous *DNAAF1* administration could restore ciliary motility in humans remains uncertain. These findings, however, provide a conceptual foundation for the development of targeted therapeutic interventions for *DNAAF1*-associated disorders, potentially complementing existing reproductive treatments.

While the genetic causes of many PCD cases remain to be elucidated, advances in genomic sequencing technologies are likely to facilitate the identification of these causative variants in the near future. *DNAAF1* variants, though relatively uncommon among PCD patients, typically disrupt the organization of both ODAs and IDAs, resulting in severe clinical symptoms. To further elucidate *DNAAF1*’s function in ciliated cells and the mechanisms involved, alternative experimental models, including organoids, may provide valuable insights.

## Data Availability

The original contributions presented in the study are publicly available. This data can be found here: https://www.ncbi.nlm.nih.gov/clinvar/ accession number SCV007496403.

## Supplementary material

SUPPLEMENTARY FIGURE S1 Uncropped scans for Figure 2C. Western blot analysis of DNAAF1 wild-type (WT) and mutant proteins expressed in HEK293 cells. Ctrl, control. IB, immunoblotting with anti-His and anti-*GAPDH* antibodies.

## References

[B1] Abramson J. Adler J. Dunger J. Evans R. Green T. Pritzel A. (2024a). Accurate structure prediction of biomolecular interactions with AlphaFold 3. Nature 630, 493–500. 10.1038/s41586-024-07487-w 38718835 PMC11168924

[B2] Abramson J. Adler J. Dunger J. Evans R. Green T. Pritzel A. (2024b). Addendum: accurate structure prediction of biomolecular interactions with AlphaFold 3. Nature 636, E4. 10.1038/s41586-024-08416-7 39604737 PMC11634763

[B3] Agarwal A. Mulgund A. Hamada A. Chyatte M. R. (2015). A unique view on Male infertility around the globe. Reprod. Biol. Endocrinol. RBE 13, 37. 10.1186/s12958-015-0032-1 25928197 PMC4424520

[B4] Basten S. G. Davis E. E. Gillis A. J. M. van Rooijen E. Stoop H. Babala N. (2013). Mutations in LRRC50 predispose zebrafish and humans to seminomas. PLoS Genet. 9, e1003384. 10.1371/journal.pgen.1003384 23599692 PMC3627517

[B5] Bracke A. Peeters K. Punjabi U. Hoogewijs D. Dewilde S. (2018). A search for molecular mechanisms underlying male idiopathic infertility. Reprod. Biomed. Online 36, 327–339. 10.1016/j.rbmo.2017.12.005 29336995

[B6] Brokaw C. J. Kamiya R. (1987). Bending patterns of Chlamydomonas flagella: IV. Mutants with defects in inner and outer dynein arms indicate differences in dynein arm function. Cell Motil. Cytoskelet. 8, 68–75. 10.1002/cm.970080110 2958145

[B7] Bustamante-Marin X. M. Ostrowski L. E. (2017). Cilia and mucociliary clearance. Cold Spring Harb. Perspect. Biol. 9, a028241. 10.1101/cshperspect.a028241 27864314 PMC5378048

[B8] Castleman V. H. Romio L. Chodhari R. Hirst R. A. de Castro S. C. P. Parker K. A. (2009). Mutations in radial spoke head protein genes RSPH9 and RSPH4A cause primary ciliary dyskinesia with central-microtubular-pair abnormalities. Am. J. Hum. Genet. 84, 197–209. 10.1016/j.ajhg.2009.01.011 19200523 PMC2668031

[B9] Duquesnoy P. Escudier E. Vincensini L. Freshour J. Bridoux A.-M. Coste A. (2009). Loss-of-function mutations in the human ortholog of Chlamydomonas reinhardtii ODA7 disrupt dynein arm assembly and cause primary ciliary dyskinesia. Am. J. Hum. Genet. 85, 890–896. 10.1016/j.ajhg.2009.11.008 19944405 PMC2790569

[B10] Faubel R. Westendorf C. Bodenschatz E. Eichele G. (2016). Cilia-based flow network in the brain ventricles. Science 353, 176–178. 10.1126/science.aae0450 27387952

[B11] Fliegauf M. Olbrich H. Horvath J. Wildhaber J. H. Zariwala M. A. Kennedy M. (2005). Mislocalization of DNAH5 and DNAH9 in respiratory cells from patients with primary ciliary dyskinesia. Am. J. Respir. Crit. Care Med. 171, 1343–1349. 10.1164/rccm.200411-1583OC 15750039 PMC2718478

[B12] Freshour J. Yokoyama R. Mitchell D. R. (2007). Chlamydomonas flagellar outer row dynein assembly protein ODA7 interacts with both outer row and I1 inner row dyneins. J. Biol. Chem. 282, 5404–5412. 10.1074/jbc.M607509200 17194703 PMC3321484

[B13] Hartill V. L. van de Hoek G. Patel M. P. Little R. Watson C. M. Berry I. R. (2018). DNAAF1 links heart laterality with the AAA+ ATPase RUVBL1 and ciliary intraflagellar transport. Hum. Mol. Genet. 27, 529–545. 10.1093/hmg/ddx422 29228333 PMC5886296

[B14] He S. Gillies J. P. Zang J. L. Córdoba-Beldad C. M. Yamamoto I. Fujiwara Y. (2023). Distinct dynein complexes defined by DYNLRB1 and DYNLRB2 regulate mitotic and male meiotic spindle bipolarity. Nat. Commun. 14, 1715. 10.1038/s41467-023-37370-7 36973253 PMC10042829

[B15] Heuser T. Raytchev M. Krell J. Porter M. E. Nicastro D. (2009). The dynein regulatory complex is the nexin link and a major regulatory node in cilia and flagella. J. Cell Biol. 187, 921–933. 10.1083/jcb.200908067 20008568 PMC2806320

[B16] Hirokawa N. Tanaka Y. Okada Y. Takeda S. (2006). Nodal flow and the generation of left-right asymmetry. Cell 125, 33–45. 10.1016/j.cell.2006.03.002 16615888

[B17] Ishibashi K. Sakakibara H. Oiwa K. (2020). Force-generating mechanism of axonemal dynein in solo and ensemble. Int. J. Mol. Sci. 21, 2843. 10.3390/ijms21082843 32325779 PMC7215579

[B18] Ito M. Morimoto K. Saotome M. Miyabayashi A. Wakabayashi K. Yamada H. (2024). Primary ciliary dyskinesia caused by homozygous DNAAF1 mutations resulting from a consanguineous marriage: a case report from Japan. Intern. Med. 63, 2847–2851. 10.2169/internalmedicine.3263-23 38432987 PMC11557207

[B19] Jayasena C. N. Sironen A. (2021). Diagnostics and management of Male infertility in primary ciliary dyskinesia. Diagnostics 11, 1550. 10.3390/diagnostics11091550 34573892 PMC8467018

[B20] Ji Z.-Y. Sha Y.-W. Ding L. Li P. (2017). Genetic factors contributing to human primary ciliary dyskinesia and Male infertility. Asian J. Androl. 19, 515–520. 10.4103/1008-682X.181227 27270341 PMC5566842

[B21] Kubo S. Yang S. K. Black C. S. Dai D. Valente-Paterno M. Gaertig J. (2021). Remodeling and activation mechanisms of outer arm dyneins revealed by cryo-EM. EMBO Rep. 22, e52911. 10.15252/embr.202152911 34338432 PMC8419702

[B22] Kumar V. Umair Z. Kumar S. Goutam R. S. Park S. Kim J. (2021). The regulatory roles of motile cilia in CSF circulation and hydrocephalus. Fluids Barriers CNS 18, 31. 10.1186/s12987-021-00265-0 34233705 PMC8261947

[B23] Lee L. Ostrowski L. E. (2021). Motile cilia genetics and cell biology: big results from little mice. Cell. Mol. Life Sci. CMLS 78, 769–797. 10.1007/s00018-020-03633-5 32915243 PMC7902362

[B24] Leung M. R. Sun C. Zeng J. Anderson J. R. Niu Q. Huang W. (2025). Structural diversity of axonemes across Mammalian motile cilia. Nature 637, 1170–1177. 10.1038/s41586-024-08337-5 39743588 PMC11779644

[B25] Liao B. Xie W. He S. (2025a). Novel heterozygous ASH1L nonsense variant involved in mild intellectual disability. Front. Neurol. 16, 1524532. 10.3389/fneur.2025.1524532 39902220 PMC11788156

[B26] Liao B. Xie W. Liu R. Zhang Q. Xie T. Jia D. (2025b). Identification of novel CDH23 heterozygous variants causing autosomal recessive nonsyndromic hearing loss. Genes Genomics 47, 293–305. 10.1007/s13258-024-01611-w 39777619 PMC11906507

[B27] Lin J. Nicastro D. (2018). Asymmetric distribution and spatial switching of dynein activity generates ciliary motility. Science 360, eaar1968. 10.1126/science.aar1968 29700238 PMC6640125

[B28] Loges N. T. Olbrich H. Fenske L. Mussaffi H. Horvath J. Fliegauf M. (2008). DNAI2 mutations cause primary ciliary dyskinesia with defects in the outer dynein arm. Am. J. Hum. Genet. 83, 547–558. 10.1016/j.ajhg.2008.10.001 18950741 PMC2668028

[B29] Loges N. T. Olbrich H. Becker-Heck A. Häffner K. Heer A. Reinhard C. (2009). Deletions and point mutations of LRRC50 cause primary ciliary dyskinesia due to dynein arm defects. Am. J. Hum. Genet. 85, 883–889. 10.1016/j.ajhg.2009.10.018 19944400 PMC2795801

[B30] Loreng T. D. Smith E. F. (2017). The central apparatus of cilia and eukaryotic flagella. Cold Spring Harb. Perspect. Biol. 9, a028118. 10.1101/cshperspect.a028118 27770014 PMC5287073

[B31] Lucas J. S. Davis S. D. Omran H. Shoemark A. (2020). Primary ciliary dyskinesia in the genomics age. Lancet Respir. Med. 8, 202–216. 10.1016/S2213-2600(19)30374-1 31624012

[B32] Lyons R. A. Saridogan E. Djahanbakhch O. (2006). The reproductive significance of human Fallopian tube cilia. Hum. Reprod. Update 12, 363–372. 10.1093/humupd/dml012 16565155

[B33] Mashiko D. Emori C. Hatanaka Y. Motooka D. Pan C. Kaneda Y. (2025). Sperm and offspring production in a nonobstructive azoospermia mouse model *via* testicular mRNA delivery using lipid nanoparticles. Proc. Natl. Acad. Sci. U. S. A. 122, e2516573122. 10.1073/pnas.2516573122 41082659 PMC12557808

[B34] Mehra B. L. Skandhan K. P. Prasad B. S. Pawankumar G. Singh G. Jaya V. (2018). Male infertility rate: a retrospective study. Urologia 85, 22–24. 10.5301/uj.5000254 28967062

[B35] Meno C. Shimono A. Saijoh Y. Yashiro K. Mochida K. Ohishi S. (1998). lefty-1 is required for left-right determination as a regulator of lefty-2 and nodal. Cell 94, 287–297. 10.1016/s0092-8674(00)81472-5 9708731

[B36] Mianné J. Ahmed E. Bourguignon C. Fieldes M. Vachier I. Bourdin A. (2018). Induced pluripotent stem cells for primary ciliary dyskinesia modeling and personalized medicine. Am. J. Respir. Cell Mol. Biol. 59, 672–683. 10.1165/rcmb.2018-0213TR 30230352

[B37] Miao C. Jiang Q. Li H. Zhang Q. Bai B. Bao Y. (2016). Mutations in the motile cilia gene DNAAF1 are associated with neural tube defects in humans. G3 6, 3307–3316. 10.1534/g3.116.033696 27543293 PMC5068950

[B38] Mitchell D. R. (2004). Speculations on the evolution of 9+2 organelles and the role of central pair microtubules. Biol. Cell 96, 691–696. 10.1016/j.biolcel.2004.07.004 15567523 PMC3321483

[B39] Munch T. N. Hedley P. L. Nielsen K. G. Christiansen M. Jurisch-Yaksi N. (2025). Exploring ciliary mechanisms in the causation of hydrocephalus in humans-similarities and differences from animal models. J. Mol. Neurosci. MN 75, 115. 10.1007/s12031-025-02405-9 40944782

[B40] Newman L. Chopra J. Dossett C. Shepherd E. Bercusson A. Carroll M. (2023). The impact of primary ciliary dyskinesia on female and Male fertility: a narrative review. Hum. Reprod. Update 29, 347–367. 10.1093/humupd/dmad003 36721921 PMC10152180

[B41] Nicastro D. Schwartz C. Pierson J. Gaudette R. Porter M. E. McIntosh J. R. (2006). The molecular architecture of axonemes revealed by cryoelectron tomography. Science 313, 944–948. 10.1126/science.1128618 16917055

[B42] O’Callaghan C. Rutman A. Williams G. M. Hirst R. A. (2011). Inner dynein arm defects causing primary ciliary dyskinesia: repeat testing required. Eur. Respir. J. 38, 603–607. 10.1183/09031936.00108410 21406509

[B43] Pazour G. J. Agrin N. Walker B. L. Witman G. B. (2006). Identification of predicted human outer dynein arm genes: candidates for primary ciliary dyskinesia genes. J. Med. Genet. 43, 62–73. 10.1136/jmg.2005.033001 15937072 PMC2593024

[B44] Pennekamp P. Menchen T. Dworniczak B. Hamada H. (2015). Situs inversus and ciliary abnormalities: 20 years later, what is the connection? Cilia 4, 1. 10.1186/s13630-014-0010-9 25589952 PMC4292827

[B45] Raidt J. Werner C. Menchen T. Dougherty G. W. Olbrich H. Loges N. T. (2015). Ciliary function and motor protein composition of human fallopian tubes. Hum. Reprod. 30, 2871–2880. 10.1093/humrep/dev227 26373788

[B46] Reiter J. F. Leroux M. R. (2017). Genes and molecular pathways underpinning ciliopathies. Nat. Rev. Mol. Cell Biol. 18, 533–547. 10.1038/nrm.2017.60 28698599 PMC5851292

[B47] Ronsard L. Ganguli N. Singh V. K. Mohankumar K. Rai T. Sridharan S. (2017a). Impact of genetic variations in HIV-1 tat on LTR-mediated transcription *via* TAR RNA interaction. Front. Microbiol. 8, 706. 10.3389/fmicb.2017.00706 28484443 PMC5399533

[B48] Ronsard L. Rai T. Rai D. Ramachandran V. G. Banerjea A. C. (2017b). *In silico* analyses of subtype specific HIV-1 Tat-TAR RNA interaction reveals the structural determinants for viral activity. Front. Microbiol. 8, 1467. 10.3389/fmicb.2017.01467 28848502 PMC5550727

[B49] Ronsard L. Sood V. Yousif A. S. Ramesh J. Shankar V. Das J. (2019a). Genetic polymorphisms in the open reading frame of the CCR5 gene from HIV-1 seronegative and seropositive individuals from national capital regions of India. Sci. Rep. 9, 7594. 10.1038/s41598-019-44136-z 31110236 PMC6527560

[B50] Ronsard L. Yousif A. S. Ramesh J. Sumi N. Gorman M. Ramachandran V. G. (2019b). *In-Vitro* subtype-specific modulation of HIV-1 trans-activator of transcription (tat) on RNAi silencing suppressor activity and cell death. Viruses 11, 976. 10.3390/v11110976 31652847 PMC6893708

[B51] Roostaei G. Khoshnam Rad N. Fakhri B M. S. Mozaffari S. Rahimi B. Kazemizadeh H. (2025). Primary ciliary dyskinesia and Male infertility: unraveling the genetic and clinical nexus. Andrology 13, 1661–1671. 10.1111/andr.13802 39572255

[B52] Saijoh Y. Adachi H. Sakuma R. Yeo C. Y. Yashiro K. Watanabe M. (2000). Left-right asymmetric expression of lefty2 and nodal is induced by a signaling pathway that includes the transcription factor FAST2. Mol. Cell 5, 35–47. 10.1016/s1097-2765(00)80401-3 10678167

[B53] Shinohara K. Kawasumi A. Takamatsu A. Yoshiba S. Botilde Y. Motoyama N. (2012). Two rotating cilia in the node cavity are sufficient to break left-right symmetry in the mouse embryo. Nat. Commun. 3, 622. 10.1038/ncomms1624 22233632

[B54] Suarez S. S. Wolfner M. F. (2021). Cilia take the egg on a magic carpet ride. Proc. Natl. Acad. Sci. U. S. A. 118, e2108887118. 10.1073/pnas.2108887118 34162738 PMC8271566

[B55] Sullivan-Brown J. Schottenfeld J. Okabe N. Hostetter C. L. Serluca F. C. Thiberge S. Y. (2008). Zebrafish mutations affecting cilia motility share similar cystic phenotypes and suggest a mechanism of cyst formation that differs from pkd2 morphants. Dev. Biol. 314, 261–275. 10.1016/j.ydbio.2007.11.025 18178183 PMC2453220

[B56] Tilley A. E. Walters M. S. Shaykhiev R. Crystal R. G. (2015). Cilia dysfunction in lung disease. Annu. Rev. Physiol. 77, 379–406. 10.1146/annurev-physiol-021014-071931 25386990 PMC4465242

[B57] van Rooijen E. Giles R. H. Voest E. E. van Rooijen C. Schulte-Merker S. van Eeden F. J. (2008). LRRC50, a conserved ciliary protein implicated in polycystic kidney disease. J. Am. Soc. Nephrol. JASN 19, 1128–1138. 10.1681/ASN.2007080917 18385425 PMC2396934

[B58] Wee W. B. Gatt D. Seidl E. Santyr G. To T. Dell S. D. (2024). Estimates of primary ciliary dyskinesia prevalence: a scoping review. ERJ Open Res. 10, 00989–02023. 10.1183/23120541.00989-2023 39104959 PMC11299005

[B59] Whitfield M. Thomas L. Bequignon E. Schmitt A. Stouvenel L. Montantin G. (2019). Mutations in DNAH17, encoding a sperm-specific axonemal outer dynein arm heavy chain, cause isolated Male infertility due to asthenozoospermia. Am. J. Hum. Genet. 105, 198–212. 10.1016/j.ajhg.2019.04.015 31178125 PMC6612517

[B60] Xie W. Liao B. Shuai M. Liu R. Hong M. He S. (2025). Novel *de novo* Intronic Variant of SYNGAP1 Associated With the Neurodevelopmental Disorders. Mol. Genet. Genomic Med. 13, e70066. 10.1002/mgg3.70066 39878419 PMC11775916

[B61] Yuan S. Liu Y. Peng H. Tang C. Hennig G. W. Wang Z. (2019). Motile cilia of the male reproductive system require miR-34/miR-449 for development and function to generate luminal turbulence. Proc. Natl. Acad. Sci. U. S. A. 116, 3584–3593. 10.1073/pnas.1817018116 30659149 PMC6397547

[B62] Yuan S. Wang Z. Peng H. Ward S. M. Hennig G. W. Zheng H. (2021). Oviductal motile cilia are essential for oocyte pickup but dispensable for sperm and embryo transport. Proc. Natl. Acad. Sci. U. S. A. 118, e2102940118. 10.1073/pnas.2102940118 34039711 PMC8179221

[B63] Yw S. L D. P L. (2014). Management of primary ciliary dyskinesia/kartagener’s syndrome in infertile male patients and current progress in defining the underlying genetic mechanism. Asian J. Androl. 16, 101–106. 10.4103/1008-682X.122192 24369140 PMC3901865

[B64] Zhou L. Li Z. Du C. Chen C. Sun Y. Gu L. (2020). Novel dynein axonemal assembly factor 1 mutations identified using whole-exome sequencing in patients with primary ciliary dyskinesia. Mol. Med. Rep. 22, 4707–4715. 10.3892/mmr.2020.11562 33174003 PMC7646867

