# Supplementary figures and images for: Exome sequencing-based identification of DNAAF1 variants implicated in male infertility and primary ciliary dyskinesia
Source: Front Mol Biosci. 2026 Apr 21;13:1769803. doi: 10.3389/fmolb.2026.1769803 (PMC13138933; doi:10.3389/fmolb.2026.1769803)

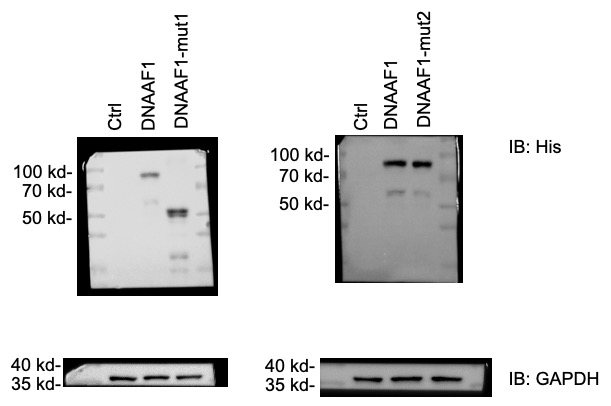

Supplement: Supplementary file 1 [file Image1.jpeg]
